# A dynamic method based on the specific substrate uptake rate to set up a feeding strategy for *Pichia pastoris*

**DOI:** 10.1186/1475-2859-10-14

**Published:** 2011-03-03

**Authors:** Christian Dietzsch, Oliver Spadiut, Christoph Herwig

**Affiliations:** 1 Vienna University of Technology, Institute of Chemical Engineering, Research Area Biochemical Engineering, Vienna, Austria

## Abstract

**Background:**

*Pichia pastoris *is one of the most important host organisms for the recombinant production of proteins in industrial biotechnology. To date, strain specific parameters, which are needed to set up feeding profiles for fed batch cultivations, are determined by time-consuming continuous cultures or consecutive fed batch cultivations, operated at different parameter sets.

**Results:**

Here, we developed a novel approach based on fast and easy to do batch cultivations with methanol pulses enabling a more rapid determination of the strain specific parameters specific substrate uptake rate q_s_, specific productivity q_p _and the adaption time (Δtime_adapt_) of the culture to methanol. Based on q_s_, an innovative feeding strategy to increase the productivity of a recombinant *Pichia pastoris *strain was developed. Higher specific substrate uptake rates resulted in increased specific productivity, which also showed a time dependent trajectory. A dynamic feeding strategy, where the setpoints for q_s _were increased stepwise until a q_s __max _of 2.0 mmol·g^-1^·h^-1 ^resulted in the highest specific productivity of 11 U·g^-1^·h^-1^.

**Conclusions:**

Our strategy describes a novel and fast approach to determine strain specific parameters of a recombinant *Pichia pastoris *strain to set up feeding profiles solely based on the specific substrate uptake rate. This approach is generic and will allow application to other products and other hosts.

## Background

Recombinant protein expression with biological hosts is one of the most examined key processes in the pharmaceutical industry. Numerous products like organic acids, antibiotics, enzymes and amino acids are produced heterologously by recombinant microorganisms. The methylotrophic yeast *Pichia pastoris *is one of the most important host organisms for this purpose.

Several of the published fermentation strategies for *P. pastoris *to date are based on the Invitrogen protocol http://tools.invitrogen.com. This protocol suggests constant feeding profiles for fed batch cultivations, but does not aim to improve production efficiency regarding time and yield or substrate consumption. Based on this protocol, different process strategies were developed to optimize recombinant protein production with *P. pastoris *in the past few years. A commonly used feeding strategy describes a feed forward regime based on a constant specific growth rate μ [1-5]. This strategy results in an exponential feeding profile and does not require complex instrumentation, but μ is also not controlled, and since the cells capacity may change over time, the feeding profiles consider a large safety margin. Another feeding strategy is based on a controlled μ and requires laborious continuous culture investigations and an effective computer controlled operation, based on established growth models and a feedback algorithm requiring expensive online measurement sensors for methanol [2,6-8]. Employing these strategies, the outcome regarding specific productivity and specific growth rate was diverse; some studies showed that the maximal specific productivity did not relate to the maximal specific growth rate [1,3,6,7], whereas another study showed a more or less growth associated productivity [9].

Due to these controversial findings, other parameters were analyzed for their possible correlation with the productivity in the past few years. Khatri and Hoffmann analyzed the specific substrate uptake rate (q_s_) and its association with the specific productivity (q_p_) in fed-batch cultivations of *P. pastoris *and showed that lower q_s _resulted in higher q_p _[10,11]. In another study, Cunha *et al*. used a constant feeding rate of methanol during several fed-batch cultivations with different initial biomass contents to monitor q_s _over time [12]. Interestingly, Cunha *et al*. also showed increased q_p _at lower q_s _and clearly stated that q_s _was the most important induction parameter. However, despite the obvious effect of q_s _on q_p_, feeding profiles based on q_s _have not been tested yet.

Regardless of which control parameter is chosen to set up feeding profiles for fed batch cultivations, strain specific parameters have to be determined. This can either be done by continuous cultures [13] or by several, consecutive fed batch cultivations, which are operated at different parameter sets [14,15]. These methods have the disadvantage of being very time-consuming and labor-intensive. A robust method to determine these strain specific data and to develop a suitable feeding strategy in a shorter time period is of high interest in biotechnology to speed up process development and to quantitatively screen industrial relevant strains.

**Figure 1:**
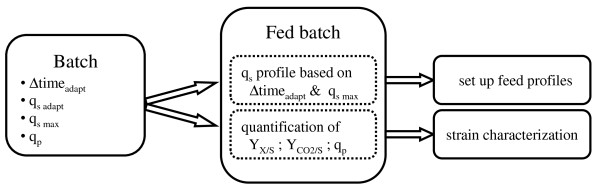
**Experimental strategy for the determination of relevant strain specific parameters in a batch cultivation with methanol pulses**. These strain specific data (Δtime_adapt _- adaptation time; q_s adapt _- specific substrate uptake rate during the adaptation period; q_s max _- maximum specific substrate uptake rate) are then used to set up a feeding profile for *P. pastoris *in fed batch cultivations. The calculated yields (Y_X/Y_, Y_CO2/S_) and the specific productivity (q_p_) can consequently be used for strain characterization.

In this study, we developed a new and fast method to determine the strain specific data q_s _and q_p_, as well as the adaptation time of the culture to the substrate methanol, based on easy to do batch cultivations with methanol pulses. The host *P. pastoris *and the product horseradish peroxidase (HRP) were used as a model system, for which we subsequently developed a novel feeding strategy solely based on the determined q_s _(Figure 1). Various fed batch cultivations, employing different q_s _feeding profiles, were carried out to characterize the strain, hence, to determine yields (Y_X/S_, Y_CO2/S_) and the specific productivity (q_p_) to evaluate and improve the feeding strategy. To our knowledge this is the first time that different dynamic feeding profiles only based on q_s _were tested to develop a feeding strategy for *P. pastoris *aiming at increased productivity.

## Material and methods

### Microorganism and recombinant protein

The *Pichia pastoris *strain KM71 H (*arg4 aox1:arg4*) was transformed with a plasmid containing the gene for the horseradish peroxidase isoenzyme C1A (HRP) and was gratefully provided by Prof. Anton Glieder (Graz University of Technology, Austria). The phenotype of the strain corresponded to an AOX1-deficient clone which is characterized as Mut^S ^(methanol utilization slow) and HRP was secreted into the fermentation broth.

Horseradish peroxidase is a member of the plant peroxidase super family [16] and catalyzes the oxidation of many substrates using hydrogen peroxide, resulting in oxidized products and in the formation of two molecules of water per molecule of hydrogen peroxide [17-19]. It is a heme-containing glycoprotein with a molecular weight of approximately 44 kDa that has been studied for more than 100 years [19]. Horseradish peroxidase gains more and more industrial relevance in the past few years; it is used in waste water treatment [20,21], analytical diagnostics [22] and for the elimination of H_2_O_2 _from food and industrial wastewater [23].

### Stability of the enzyme horseradish peroxidase in the presence of methanol

To check whether certain concentrations of methanol were affecting the stability of the enzyme horseradish peroxidase, HRP was diluted either in water or in BSM medium to a concentration of 1 U/mL and incubated in a waterbath at 28°C in the presence of up to 20 mg/mL methanol (which corresponds to 2.5% v/v) overnight. At several time points samples were taken and analyzed for catalytic activity and protein content.

### Culture Media

Preculture: Yeast nitrogen base media (YNBM), per liter: potassium phosphate buffer (pH 6.0), 0.1 M; YNB w/o Amino acids and Ammonia Sulfate (Difco™), 3.4 g; (NH_4_)_2_SO_4_, 10 g; biotin, 400 mg; glucose, 20 g.

Batch/fed batch: Basal salt media (BSM) [24], per liter: 85% phosphoric acid, 26.7 mL; CaSO_4_·2H_2_0, 1.17 g; K_2_SO_4_, 18.2 g; MgSO_4_·7H_2_O, 14.9 g; KOH, 4.13 g; C_6_H_12_O_6_·H_2_O, 44 g, Antifoam Struktol J650, 0.2 mL; PTM1, 4.35 mL; NH_4_OH as N-source (see experimental procedure). Trace element solution (PTM1), per litre: CuSO_4_·5H_2_O, 6.0 g; NaI 0.08 g; MnSO_4_·H_2_O, 3.0 g; Na_2_MoO_4_·2H_2_O, 0.2 g; H_3_BO_3_, 0.02 g; CoCl_2_, 0.5 g; ZnCl_2_, 20.0 g; FeSO_4_·7H_2_O, 65.0 g; biotin, 0.2 g, H_2_SO_4_, 5 mL.

Feed glucose, per liter: glucose, 250 g; PTM1, 12 mL, Struktol J650, 0.3 mL.

Feed methanol, per liter: methanol, 300 g; PTM1, 4 mL; Struktol J650, 0.3 mL, induction period was carried out in presence of δ-Aminolevulinic acid (δ-ALA), 1 mM.

Base: NH_4_OH, concentration was determined by titration with 0.25 M potassium hydrogen phthalate (KHP).

### Experimental Procedure

#### Preculture

Frozen stocks (-80°C) were pre-cultivated in 100 mL of YNBM in 1000 mL shake flasks at 28°C and 200 rpm for max. 24 hours. Then, the preculture was transferred aseptically to the respective culture vessel. The inoculation volume was approximately 10% of the final starting volume.

#### Batch cultivation and determination of q_s_

Batch cultivations were carried out in a 1 L working volume glass bioreactor (Applikon, Netherlands). Basal salt media was sterilized in the bioreactor and pH was adjusted to pH 5.0 by using concentrated ammonia solution after autoclaving. Sterile filtered trace elements were transferred to the reactor aseptically. Dissolved oxygen (dO_2_) was measured with a sterilizable polarographic dissolved oxygen electrode (Mettler Toledo, Switzerland). The pH was measured with a sterilizable electrode (Mettler Toledo, Switzerland) and maintained constant with a step controller using ammonia solution (1 to 2 M). Base consumption was determined gravimetrically. Cultivation temperature was set to 28°C and agitation was fixed to 1200 rpm. The culture was aerated with 1.25 vvm dried air and off-gas of the culture was measured by using an infrared cell for CO_2 _and a paramagnetic cell for O_2 _concentration (Servomax, Switzerland). Temperature, pH, dO_2_, agitation as well as CO_2 _and O_2 _in the off-gas were measured online and logged in a process information management system (PIMS; Lucullus, Biospectra, Switzerland).

**Figure 2:**
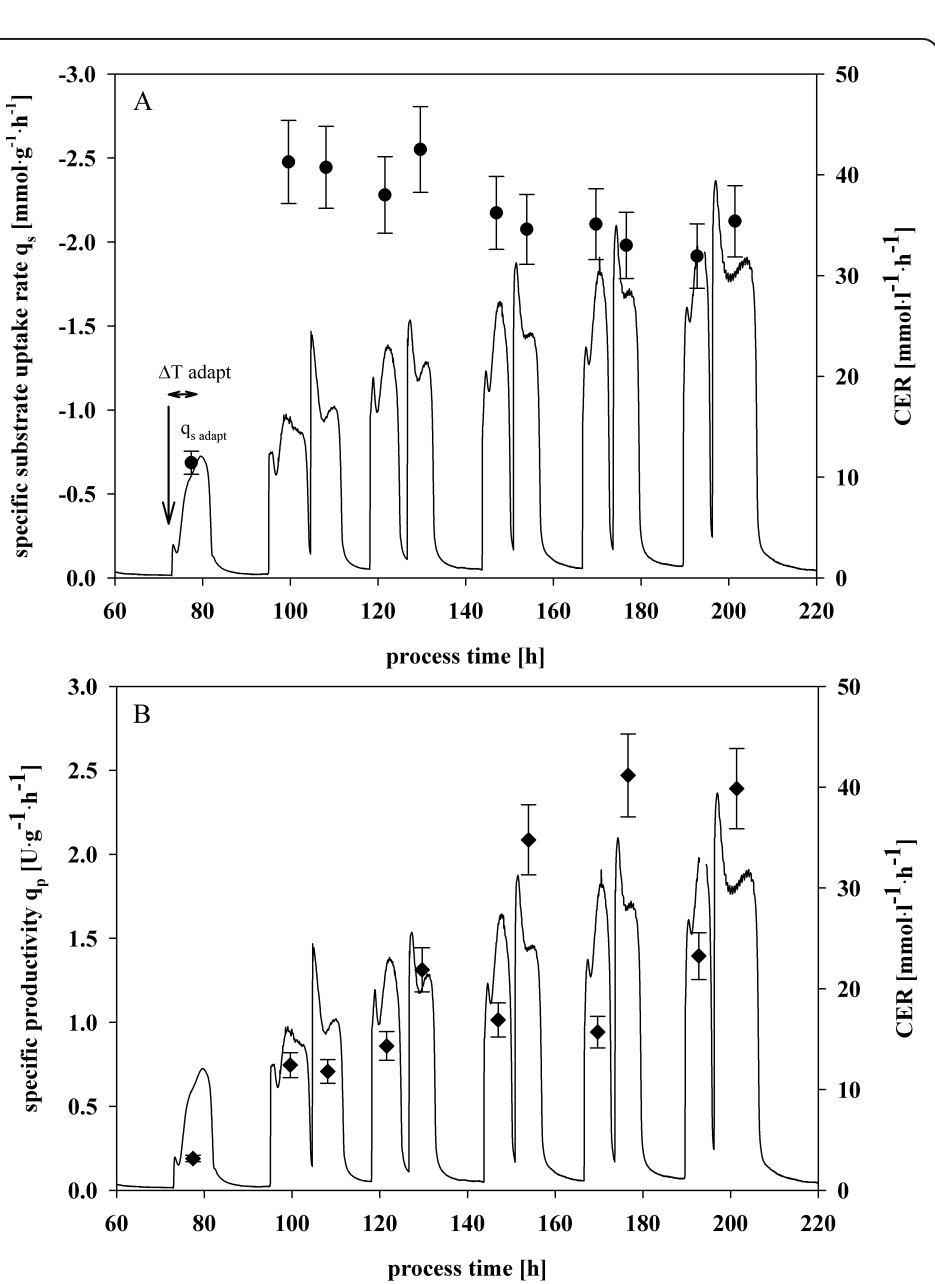
**Batch cultivation with methanol pulses of 0.5, 1 and 2% (v/v)**. A, calculated specific substrate uptake rate q_s _(circle) and carbon dioxide evolution rate (CER); B, specific production rate q_p _(diamond) and carbon dioxide evolution rate (CER).

After the complete consumption of the substrate glucose, which was indicated by an increase of dissolved oxygen and a drop in off-gas activity, the first methanol pulse of a final concentration of 0.5% (v/v) was conducted with pure methanol (supplemented with PTM1, 12 mL/L of methanol). Following pulses were performed with 1% (v/v), before a last pulse with 2% (v/v) final concentration of methanol was carried out. To investigate the metabolic activity during methanol excess and also the dynamic behavior of the cell metabolism after methanol limitation for several hours, the pulse experiments were performed like this: after methanol was depleted after the "first" pulse (followed by off-gas analysis), an immediate "second" methanol pulse with the same concentration was conducted per day. After methanol depletion, methanol starvation was carried out for several hours before another so-called "first" pulse was applied (Figure 2).

For each pulse, at least two samples were taken to determine the concentrations of substrate and product as well as dry cell weight and OD_600 _to calculate the specific substrate uptake rate q_s_.

#### Fed batch cultivations

Fed batch cultures were carried out in a 7.5 L (5 L working volume) glass bioreactor (Infors, Switzerland). Concentrated BSM medium (2-fold concentrated to supply necessary salts for high cell densities) was sterilized in the bioreactor and pH was adjusted to pH 5.0 by using concentrated ammonia solution after autoclaving. The initial volume was set to 1.5 L. Trace elements were filter sterilized and transferred to the reactor aseptically. Dissolved oxygen was measured with a sterilizable polarographic dissolved oxygen electrode (Hamilton, Switzerland). The pH was measured with a sterilizable electrode (Hamilton, Switzerland) and maintained constant using ammonia solution (3 to 5 M). Agitation was set to 1500 rpm. The culture was aerated with at least 1 vvm to avoid oxygen limitation. The dissolved oxygen signal was used to adjust air-in flow manually to keep levels >30% dO_2 _at all time points. In case air flow was limited, pure oxygen was added. CO_2 _and O_2 _were measured as described above. Base consumption and reactor weight were measured gravimetrically. The fed batch feed was measured and controlled using a gravimetrically based PID flow controller.

At several time points during fed batch cultivations, samples were taken and analyzed for accumulated methanol, biomass concentration (dry cell weight and optical density OD_600_), protein content and enzymatic activity. Based on the total biomass content, feed rates were adjusted manually corresponding to the defined q_s _setpoint.

Three different fed batch strategies were tested: fed batch A, where methanol was adjusted to a constant flow during the whole induction phase; fed batch B, where after the adaption time with a q_s adapt_, a shift to a high substrate uptake rate of ~90% of q_s max _was done and adjustments to the very same q_s _set point during cultivation were performed repeatedly; and fed batches C1 and C2, where after the adaptation period with q_s adapt_, the methanol flow was stepwise increased up to q_s max_. Values for q_s adapt _and q_s max _had been determined in batch experiments (*vide supra*). An overview of the fed batch cultivations and the corresponding settings is given in Table 1.

**Table 1:** Description of feeding strategies for fed batch cultivations based on the specific substrate uptake rate q_s_.

Fed batch name	Symbol	Description of the strategy
Fed batch A	**▲**	conventional feeding strategy: long adaptation time and initially adjusted, constant flow rate
Fed batch B	◆	short adaptation time (out of batch exp.) and adjustment to high uptake rate (90% of q_s max_) with repeated readjustments
Fed batch C1 Fed batch C2	**▼** ** ●**	short adaptation time (out of batch exp.) and stepwise adjustment of q_s _until q_s max _with repeated readjustments

### Analysis of growth- and expression-parameters

Dry cell weight (DCW) was determined by centrifugation of 5 mL culture broth (5000 rpm, 4°C, 10 min) in a laboratory centrifuge (Sigma 4K15, rotor 11156), washing the pellet with 5 mL deionized water and subsequent drying at 105°C to a constant weight in an oven. Optical density of the culture broth was measured using a spectrophotometer (U-1100 Hitachi, Japan) at a wavelength of 600 nm (OD_600_). Correlation between dry cell weight measurement and OD_600 _showed a coefficient of regression of R^2 ^= 0.997 over the full concentration range (data not shown) and could therefore be used for q_s _adaptation.

The activity of HRP was determined using a CuBiAn XC enzymatic robot (Innovatis, Germany). Cell free samples (10 μl) were added to 140 μl of 1 mM ABTS (2.2' azino bis 3-ethylbenzthiazoline-6-sulphonic acid) prepared in 50 mM NaOAc buffer (pH 4.5). The reaction mixture was incubated at 37°C and was started by the addition of 20 μl of 0.075% H_2_O_2_. Changes of absorbance at 415 nm were measured for 80 seconds and rates were calculated. Calibration was done using commercially available horseradish peroxidase (Type VI-A, Sigma-Aldrich, P6782, Lot# 118K76703) as standard at six different concentrations (0.02; 0.05; 0.1; 0.25; 0.5 and 1.0 U/mL). Samples with high enzymatic activity were automatically diluted by the system. Protein concentrations were determined at 595 nm by the Bradford assay [25] using the BioRad Protein Assay Kit with BSA as standard.

### Substrate concentrations

Concentrations of methanol were determined in cell free samples by HPLC (Agilent Technologies, USA) equipped with a Supelcoguard column, a Supelcogel C-610 H ion-exchange column (Sigma-Aldrich, USA) and a refractive index detector (Agilent Technologies, USA). The mobile phase was 0.1% H_3_PO_4 _with a constant flow rate of 0.5 mL/min and the system was run isocratic. Calibration was done by measuring standard points in the range of 0.1 to 10 g/L methanol.

Concentrations of glucose were determined in cell free samples by a commercial enzymatic assay kit using the CuBiAN XC enzymatic robot (Innovatis, Germany). Calibration was done with 4 standard points in the range from 0 to 3 g/L glucose. Samples with higher glucose concentration were diluted automatically by the system.

### Data analysis

Measurements of biomass concentration, product concentration and substrate concentration were executed in duplicates: along the observed standard deviation for the single measurement, the error was propagated to the specific rates q_s _and q_p _as well as to the yield coefficients. The error of determination of the specific rates and the yields was therefore set to 10% and 5%, respectively.

### Electrophoresis

To check the purity of the excreted HRP, electrophoresis was done with aliquots of supernatants obtained at different time points during the cultivation of *P. pastoris *expressing the *hrp *gene extracellularly as described by Laemmli *et al. *[26]. SDS-PAGE was performed using a 5% stacking gel and a 10% separating gel in 1× Tris-glycine buffer. Gels were run in the vertical electrophoresis Mini-PROTEAN Tetra Cell apparatus (Biorad; Vienna, Austria) at 150 V for about 2 h. Gels were stained with Coomassie blue. The protein mass standard used was the PageRuler Prestained Ladder (Fermentas; Vienna, Austria).

## Results and Discussion

### Stability of the enzyme horseradish peroxidase in the presence of methanol

To check whether the enzyme horseradish peroxidase denatures at certain concentrations of methanol, HRP was incubated in the presence of methanol concentrations of up to 2.5% (v/v) at 28°C overnight. No loss in catalytic activity and protein content was detected after this incubation, which is why methanol pulses in batch experiments with concentrations of up to 2.5% (v/v) were theoretically possible without any denaturing effects on HRP.

### Determination of strain specific parameters by easy to do batch cultivations with methanol pulses

After depletion of glucose in batch cultivations, a first methanol adaption pulse with a final concentration of 0.5% (v/v) was applied. The adaptation time to the new substrate methanol (Δtime_adapt_) was determined with 7 hours and was defined by the detection of a maximum in off-gas activity (Figure 2A).

The calculated carbon dioxide evolution rate (CER), signifying metabolic activity, allowed to distinguish different states within the methanol pulses with local minima and maxima (Figure 2). This metabolic behavior of the cells results from inhibition and regulation events in transient conditions, caused by intracellular components like e.g. produced H_2_O_2_, and transport actions, and has been described for various systems in literature before [27-29].

The high frequent determination of biomass, methanol and product concentrations allowed specific rate calculations for methanol uptake q_s _and productivity q_p _during the methanol pulses. Specific substrate uptake rates were calculated with 0.8 ± 0.08 mmol·g^-1^·h^-1 ^in the adaptation period (q_s adapt_) and with around 2 ± 0.20 mmol·g^-1^·h^-1 ^as a maximum during pulses (q_s max_). No difference in the calculated q_s _between the "first" and the subsequent "second" pulse on a respective day was observed (Figure 2A). In contrast, the calculated values for q_p _(Figure 2B) were very different between the first and the subsequent second pulse. During the first pulse specific productivities of maximum 1.3 ± 0.13 U·g^-1^·h^-1 ^were observed, whereas during the second pulse an increased specific productivity of up to 2.5 ± 0.25 U·g^-1^·h^-1 ^was measured. This increased productivity during the second methanol pulse may be due to the fact that no adaption of the cells to methanol was necessary because all metabolic key functions for methanol assimilation remained in an active state. Thus, recombinant protein expression could start directly and energy was used more efficiently for product formation. This result shows clearly that *Pichia *cultures should be kept induced at all time to obtain maximal productivity. Interestingly, q_p _also increased over time for first and second pulses, respectively (Figure 2B). Methanol pulses, where the same final concentration of methanol was used, but which were conducted in the later phase of the batch cultivation, showed higher specific productivities compared to pulses before. Obviously, the culture exhibited a "memory effect" and thus a time-dependence of q_p _could be observed in the batch experiment.

As we show here, batch cultivations with methanol pulses allowed a fast identification of strain specific parameters, which are crucial for subsequent fed batch cultivations. The determined maximum specific substrate uptake rate q_s max_, represents the upper end of the feed profile respective to q_s_. This novel method has the advantage of being less time-consuming and labor-intensive compared to the traditional methods, like continuous cultivations, and additionally allows a free choice of substrate, like e.g. the 2 to 3-fold cheaper glucose instead of glycerol.

### Fed batch feeding strategy based on q_s_

Based on the batch results, we performed several fed batch cultivations with different feeding profiles based on the specific substrate uptake rate q_s_, to find a feeding strategy for a recombinant *P. pastoris *strain.

#### Feeding profiles

After a batch phase on glucose as substrate (volume 1.5 L), an exponential fed batch cultivation with glucose yielded in biomass concentrations of up to 70 g/L in a volume of 2.5 L. At the end of this fed batch phase, a sample was taken to determine the current biomass concentration by measuring the OD_600 _and the DCW.

**Figure 3:**
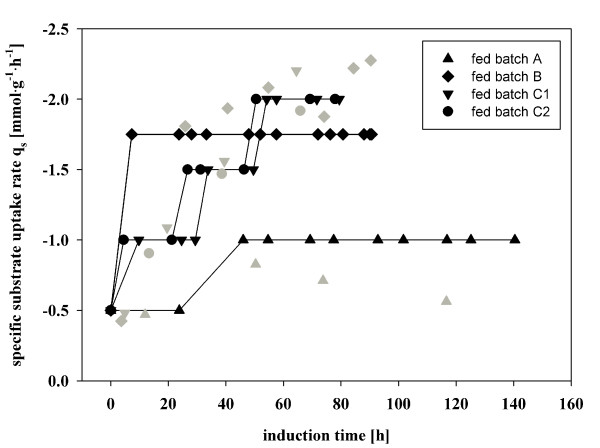
**Specific substrate uptake rate profiles which were used for different fed batch cultivations**. Theoretical q_s _fed batch A (black triangle up), offline determined q_s _fed batch A (grey triangle up); theoretical q_s _fed batch B (black diamond), offline determined q_s _fed batch B (grey diamond); theoretical q_s _fed batch C1 (black triangle down), offline determined q_s _fed batch C1 (grey triangle down); theoretical q_s _fed batch C2 (black circle), offline determined q_s _fed batch C2 (grey circle). The error of determination for offline q_s _(10%) is not shown for better readability.

Based on the calculated specific methanol uptake rate during adaptation (q_s adapt_) from the batch experiment, which was around 0.8 ± 0.08 mmol·g^-1^·h^-1^, we used a little bit lower q_s adapt _of 0.5 mmol·g^-1^·h^-1 ^methanol for the fed batch cultivations during the adaption time. After Δtime_adapt_, three different feeding strategies (fed batches A, B and C1/C2) were tested and compared (Figure 3 Table 1).

For fed batch A, which described a conventional feeding strategy as in the Invitrogen protocol, the methanol feed was adjusted to a substrate uptake rate of 1.0 mmol·g^-1^·h^-1^. The adjustment just happened at the beginning and no further readjustments of the feeding rate were performed during the cultivation. Consequently, as biomass still increased, a drop down of q_s _over time was observed (Figure 3).

In the other fed batch experiments B, C1 and C2 adjustments of q_s _were done after each sampling, based on OD_600 _measurements. As shown in Figure 3 the effective q_s_, determined by off-line sampling, and the designated q_s _in these fed batch cultivations were very similar. Thus, a frequent determination of the actual biomass concentration in the reactor vessel is necessary, to be able to adjust the feeding rate to the chosen q_s_. The development of robust measurement devices for the online determination of the biomass concentration, as e.g. FTIR, capacity probes or soft sensors, could allow an online adjustment of the feeding rate to q_s _by an automatic control system in the future, and thus the labor-intensive, frequent sampling procedure would be unnecessary.

#### Maintenance metabolism vs. cell growth

**Figure 4:**
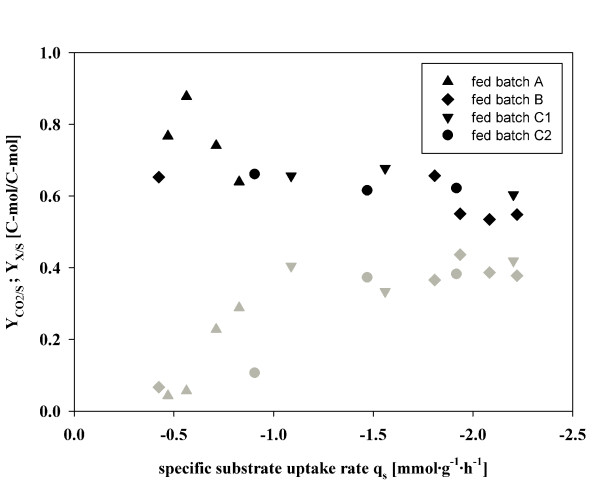
**Carbon dioxide yield (Y_CO2/S_) and biomass yield (Y_X/S_) at different specific substrate uptake rates in fed batch cultivations**. Y_CO2/S _fed batch A (black triangle up), Y_X/S _fed batch A (grey triangle up); Y_CO2/S _fed batch B (black diamond), Y_X/S _fed batch B (grey diamond); Y_CO2/S _fed batch C1 (black triangle down), Y_X/S _fed batch C1 (grey triangle down); Y_CO2/S _fed batch C2 (black circle); Y_X/S _fed batch C2 (grey circle). The error of determined yields (5%) is not shown for better readability.

The relationship between q_s _and the specific growth rate was tested and found to be linearly correlated (data not shown). Based on this, the maintenance coefficient for this *P. pastoris *strain could be determined with around 0.5 mmol·g^-1^·h^-1 ^(which equals 0.016 g·g^-1^·h^-1^). Very similar results were reported by Jahic *et al*. for fed batch cultures using *P. pastoris *expressing a fusion protein [5] with a maintenance coefficient of 0.013 g·g^-1^·h^-1^. When we adjusted q_s _to values higher than 1.0 mmol·g^-1^·h^-1^, we observed a significant increase in Y_X/S _and a corresponding decrease in Y_CO2/S _(Figure 4). That means that at q_s _of <1.0 mmol·g^-1^·h^-1 ^most of the energy is obviously used for maintenance metabolism of the cells and thus for the production of CO_2 _and not for cell growth or protein production. Interestingly, the same effect on biomass yield was observed by d'Anjou *et al*. using a continuous culture system with a mixed feed strategy [13]. However, we were able to extract this information from time-efficient, dynamic experiments equivalently. Consequently, we employed specific substrate uptake rates of >1.0 mmol·g^-1^·h^-1 ^to guarantee a sufficient amount of energy for both, maintenance and cell growth.

#### Required methanol concentration for induction - a principle question

When producing recombinant proteins with *P. pastoris*, the principle question is how much methanol has to be fed to guarantee a fully induced AOX promoter. A lot of different studies have examined this topic, with different results. D'Anjou *et al*., for example, reported that a methanol concentration between 1 and 2 g/L should be maintained in the culture to guarantee fully induced heterologous protein production [13]. Another study of the methanol concentration and its effect on *P. pastoris *Mut^S ^strains was done by Kupcsulik and Sevella, who showed that the specific productivity of a recombinant human serum albumin expression system showed a maximum at 0.45 g/L of methanol [30]. In contrast to those studies, Cregg reported an even stronger induction of the AOX promoter in limited conditions [31], which was the basis for several following studies applying a limited methanol supply for induction [1-3].

For all described fed batch strategies in our study, no significant methanol accumulation was detected (detection limit 0.1 g/L). We clearly show that high residual concentrations of methanol during the production phase are not required for the induction of the *P. pastoris *Mut^S ^strain, as even higher specific productivities were obtained in limited fed batches with constant substrate uptake rates, compared to the batch cultivations with high methanol concentrations applied in the pulse-experiments (Table 2).

**Table 2:** Comparison of the specific substrate uptake rates q_s _and determined maximum specific productivities q_p_.

**Experiment**^**a**^	Methanol concentration	**Specific substrate uptake rate q**_**s **_**[mmol·g**^**-1**^**·h**^**-1**^**]**	**Specific productivity q**_**p **_**[U·g**^**-1**^**·h**^**-1**^**]**
Batch with methanol pulses	from 0 to 16 g/L	~ 2	2.5 ± 0.25
Fed batch A ▲	limited	< 1	2.0 ± 0.20
Fed batch B ◆	limited	1.75	5.0 ± 0.50
Fed batch C1 ▼	limited	stepwise up to 2	10 ± 0.10
Fed batch C2 **●**	limited	stepwise up to 2	11 ± 0.11

^a ^all experiments were performed in the presence of different concentrations of the inducer methanol

#### Extracellular protein production and specific productivity q_p_

**Figure 5:**
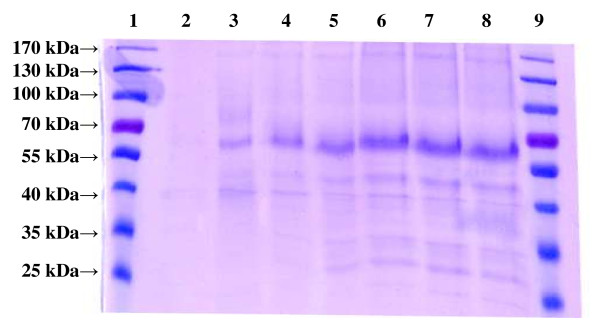
**SDS-PAGE analysis of recombinantly produced HRP in fed batch B**. Aliquots of the supernatant obtained at different time points during the cultivation of *P. pastoris *expressing the *hrp *gene extracellularly at 28°C were loaded onto the gel. lanes 1 and 9, molecular mass standard; lane 3, not induced (after fed-batch phase with glucose); lane 3-8, 7 h; 33 h; 58 h; 76 h; 88 h; 91 h of induction.

To follow the formation of excreted horseradish peroxidase (HRP) during the induction phase, SDS-PAGE analyses with cell-free supernatants were performed. SDS-PAGE analysis (Coomassie staining, Figure 5) of the cultivation broth taken at different time points did not show a distinct band for HRP, but rather a smear between 60 - 65 kDa. The difference between the reported molecular weight of around 44 kDa for HRP and the observed molecular weight in this study results from the high degree of glycosylation of the recombinant protein expressed in *P. pastoris *with mannose-type oligosaccharides, as described previously for HRP by Morawski *et al. *[32].

The protein content in the cultivation broth increased over time from 0.052 mg/mL after the fed-batch phase with glucose to 0.243 mg/mL at the end of the cultivation, which were around 91 hours of induction. As shown in Figure 5 the increase of the protein content in the cultivation broth is ascribed to an increased amount of HRP, which constituted the majority of secreted proteins in the cultivation broth.

**Figure 6:**
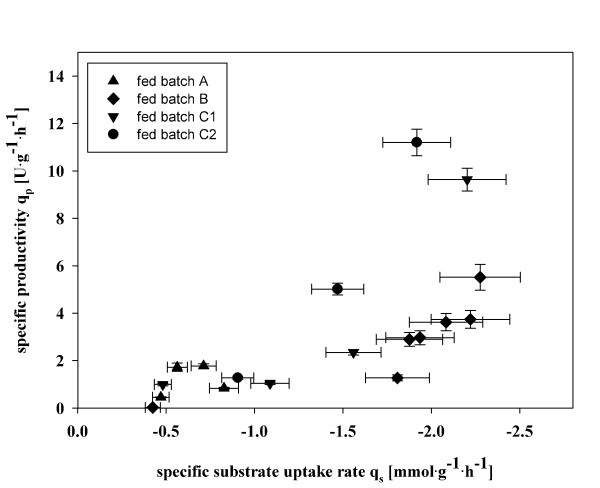
**Specific productivity (q_p_) plotted against the specific substrate uptake rate (q_s_)**. Fed batch A (triangle up); fed batch B (diamond); fed batch C1 (triangle down); fed batch C2 (circle).

Based on the determined biomass content and the enzymatic activity, the specific productivity q_p _during the different fed batch cultivations was calculated. The lowest q_p _was obtained in fed batch A, which described a conventional feeding strategy, where also the lowest q_s _was used and was not adapted over time (Figure 6). In fed batch B, which considered the actual biomass concentration and where the q_s _of 1.75 mmol·g^-1^·h^-1 ^was adapted regularly over time and thus the cells were kept in a certain physiological state, a 2-fold increase in q_p _was observed. Clearly, a higher q_s _resulted in a higher q_p_, which disagrees with the results obtained by Khatri and Hoffmann and Cunha *et al. *[10,11], who stated that lower q_s _resulted in higher protein production.

However, a dynamic, stepwise feeding strategy resulted in an even higher productivity compared to the other strategies tested (Figure 6). This dynamic feeding strategy considered q_s max_, which had been determined in the batch experiment, as the highest possible substrate uptake rate of the cells, as well as the yield coefficients and the maintenance coefficient as the lower end of efficient energy usage. The feed profile was set up in a way to head off as quickly as possible from the maintenance state of the cells (hence q_s _was set to values ≥1.0 mmol·g^-1^·h^-1^), before q_s _was dynamically adapted in steps to q_s max_. In fed batch cultivations C1 and C2, where this dynamic feeding strategy was applied, a q_p _of around 11 ± 0.11 U·g^-1^·h^-1 ^was determined, which represents a 5.5-fold increase compared to fed batch A.

#### Time adaptation

**Figure 7:**
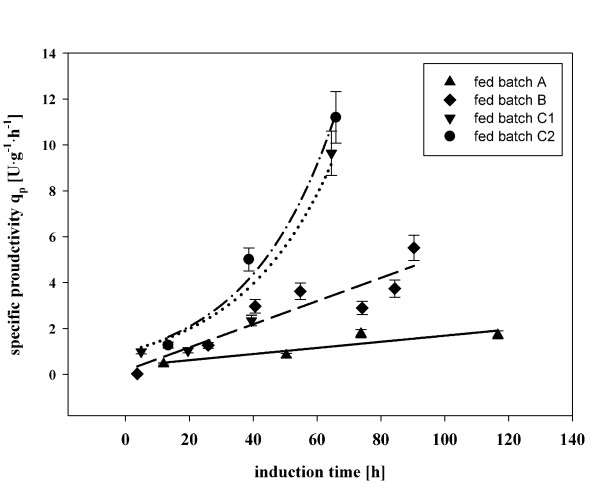
**Specific productivity (q_p_) in fed batch cultures over the induction time**. Fed batch A (triangle up); fed batch B (diamond); fed batch C1 (triangle down); fed batch C2 (circle). Regression lines: linear regression for fed batch A (continuous line); linear regression for fed batch B (discontinuous line); exponential regression for fed batch C1 (dotted line); exponential regression for fed batch C2 (dashed-dotted line).

The fact that there was still a more than 2-fold difference in q_p _between fed batches C1/C2, where q_s _was increased stepwise, and fed batch B, where immediately after the adaption period a high q_s _of 1.75 mmol·g^-1^·h^-1 ^was applied, indicated another factor being crucial for the specific productivity besides q_s_. Therefore, the specific productivity q_p _was plotted against the induction time, to analyze possible time-dependent effects (Figure 7). Fed batch A and B were characterized by a linear increase of q_p _over time (fed batch B was characterized by a significant steeper slope), whereas fed batches C1 and C2 even showed an exponential increase of the specific productivity. It became obvious that the productivity was directly correlated to the induction time for all experiments conducted in this study, even in the batch experiments (Figure 2). Plantz *et al*. have recently reported an influence of the induction time on q_p _as well. They showed increasing product yields for the recombinant production of an interferon with *P. pastoris *in the first period of induction, but a subsequent decrease in product formation and a shift of the energy transfer towards biomass growth during the later phase of the cultivation [8]. In contrast to the study of Plantz *et al*., we clearly showed an increase of q_p _over the whole induction time for all experiments in this study (Figure 7).

Furthermore, fed batches C1 and C2 were characterized by an exponential increase of q_p _over time, in contrast to a linear increase for fed batches A and B (Figure 7). Obviously, a feeding strategy starting with a lower q_s _at the beginning of the induction phase, followed by a subsequent increase with a stepwise, dynamic feeding profile until q_s max _(fed batch C1 and C2), was superior to a feeding strategy, where immediately after the Δtime_adapt _a high q_s _was applied and kept constant (fed batch B). When the cells had the time to undergo a dynamic, physiological adaptation to the current culture condition before q_s _was increased, a boost in q_p _was observed, which probably cannot be triggered by constant or exponential feeding regimes (Table 2).

Summarizing, we showed a clear time dependent trajectory for specific product formation, as q_p _increased over induction time for all experiments in this study. A tool for the early identification of this effect is of utmost importance in order to consistently compare different feeding profiles.

## Conclusions

In this study, we developed a fast and easy to do method based on batch cultivations with methanol pulses to determine strain specific parameters of a *P. pastoris *Mut^S ^strain expressing the enzyme horseradish peroxidase. A subsequently developed dynamic feeding strategy solely based on q_s_, where the cells on the one hand had time to adapt to culture conditions, but were then challenged again repeatedly by a stepwise increase of q_s _up to q_s max_, resulted in the highest q_p _compared to the other strategies tested. Hence, dynamic feeding profiles turned out to be a valuable method to boost the specific productivity. This calls for increased use of dynamic process conditions even for industrial feed profiles. We strongly believe that the strategy presented here can be successfully applied on other microbial expression systems, which is why we are currently testing the applicability of our novel, dynamic approach on other expression systems, like *E. coli*, and other products.

## Abbreviations

Δtime_adapt_: time for adaptation of the culture to the new substrate (methanol) [h]; μ specific growth rate [h^-1^]; CER: carbon dioxide evolution rate [mmol·L^-1^·h^-1^]; HRP: horseradish peroxidase; Mut^S^: methanol utilization slow phenotype; PID: proportional-integrative-derivative controller; q_p_: specific productivity of horseradish peroxidase [U·g^-1^·h^-1^]; q_s_: specific substrate uptake rate [mmol·g^-1^·h^-1^]; q_s adapt_: specific substrate uptake rate during adaptation [mmol·g^-1^·h^-1^]; q_s max_: maximum specific substrate uptake rate [mmol·g^-1^·h^-1^]; rpm: rounds per minute; vvm: volume gas flow per volume liquid per minute; Y_CO2/S_: yield coefficient of carbon dioxide respective to methanol [C-mol·C-mol^-1^]; Y_X/S_: yield coefficient of biomass respective to methanol [C-mol·C-mol^-1^]

## Competing interests

The authors declare that they have no competing interests.

## Authors' contributions

CD designed and performed the experiments and analyzed data. OS performed some experiments. CD and OS wrote the paper, CH conceived the study and supervised research. All authors read and approved the final manuscript.
